# Ischemic heart disease awareness in Egypt's aging population: findings from a national cross-sectional study

**DOI:** 10.1186/s43044-024-00584-1

**Published:** 2024-11-22

**Authors:** Mohamed Saad Rakab, Mohamed Baklola, Basel Hatem Elsalakawi, Moaz Mohamed Zaki, Mohamed Elhusseini Elsaeidi, Hossam Fouad, Belal Walid, Abdelrahman Elbaz, Ali Emara, Abdelrahman Sherif Ghanem, Nada Mohammed Radwan, Ahmed Reda Abdelmeguid, Eman Elsayed Alkalla, Rehab Shaheen Bahram Shaheen

**Affiliations:** 1 Faculty of Medicine, Mansoura University, Mansoura, Egypt; 2 Internal Medicine and Critical Care Unit, Mansoura University, Mansoura, Egypt

**Keywords:** Ischemic heart disease, Elderly population, Awareness, Risk factors, Warning signs, Egypt, Public health

## Abstract

**Background:**

Ischemic heart disease (IHD) remains a leading cause of mortality among the elderly population, particularly in low- and middle-income countries like Egypt, where public health infrastructure may struggle to meet the needs of a growing aging population. Awareness of the risk factors and warning signs associated with IHD is critical for early detection and intervention. This study aimed to evaluate the level of awareness and knowledge regarding IHD risk factors and warning signs among elderly individuals in Egypt, focusing on differences between urban and rural populations.

**Results:**

Among 595 participants aged 60 years and above, only 18.7% demonstrated good knowledge of IHD risk factors, whereas 47.2% were categorized as having poor knowledge. In terms of warning signs, 7.2% exhibited good awareness, while 47.9% showed poor awareness. Overall, more than half (51.8%) of the participants were found to have poor knowledge of IHD, and just 15.1% had good overall awareness of both risk factors and warning signs. Urban participants exhibited significantly higher knowledge compared to rural participants (*p* < 0.05). Educational attainment was a strong predictor of knowledge, with participants holding postgraduate degrees scoring the highest. Economic status also influenced awareness, with those in the excellent category demonstrating significantly higher knowledge (*p* < 0.05). Occupation had a notable impact, with engineers exhibiting the highest levels of awareness and farmers the lowest. Gender, however, was not a significant factor, with males and females showing similar levels of awareness.

**Conclusions:**

The study reveals a significant knowledge gap concerning IHD risk factors and warning signs among Egypt’s elderly population, particularly in rural areas and among individuals with lower levels of education and economic status. This gap underscores the need for targeted public health campaigns and interventions, particularly in rural regions, to raise awareness and reduce the burden of IHD among Egypt's elderly. Enhanced education and community-based programs could be effective in mitigating the risks associated with poor awareness of IHD.

**Supplementary Information:**

The online version contains supplementary material available at 10.1186/s43044-024-00584-1.

## Background

Cardiovascular disease (CVD) remains the leading cause of death worldwide, accounting for 17.3 million deaths annually—a figure projected to exceed 23.6 million by 2030 [1, 2]. The burden of CVD is disproportionately high in low- and middle-income countries, which account for 80% of these deaths, particularly in the Eastern Mediterranean Region (EMR) [2, 3]. In the EMR, non-communicable diseases, including CVD, contribute to 34% of all fatalities, with ischemic heart disease (IHD) being a predominant cause [3].

Over the past three decades, the prevalence of CVD in the EMR has been driven by significant lifestyle changes and the increasing prevalence of risk factors such as smoking, obesity, hypertension, diabetes, and physical inactivity [3, 4]. In Egypt, studies have shown that reducing exposure to these risk factors could substantially decrease CVD-related mortality, with potential reductions of 9.5% for smoking, 4.1% for obesity, 11% for hypertension, 8.2% for diabetes, and 5.4% for overweight [5].

Previous studies have demonstrated that socioeconomic factors significantly influence health literacy. Probably the most important of which is advanced age, where elderly showed more limited knowledge about stroke and heart attack symptoms, as well as cardiovascular disease risk factors [6]. In another study exploring CVD knowledge among elderly, 56.9% of 1120 participants had poor understanding about CVD, compared to only 0.8% who had strong knowledge [7].

Despite the critical importance of awareness in preventing CVD, studies have indicated that populations in the EMR, including Egypt, remain inadequately informed about the role of risk factors in the development of these diseases [5]. For example, a study in Jordan revealed that a significant proportion of the population lacked sufficient knowledge about CVD​, and a similar study in Syria found that only 61.5% of participants were adequately aware of CVD risk factors [8, 9].

However, there is a notable gap in research specifically addressing the awareness of ischemic heart disease among Egypt's aging population—a demographic that is particularly vulnerable to IHD. Understanding the level of awareness in this group is essential for developing targeted public health interventions aimed at reducing the incidence and impact of ischemic heart disease.

## Methods

### Study design

This study employed a cross-sectional design to assess the awareness and knowledge of ischemic heart disease (IHD) risk factors and warning signs among the elderly population in Egypt. Data were collected over a two-week period from May 7 to May 21, 2024. The study targeted individuals aged 60 years and above, residing in both urban and rural areas across Egypt. The primary objective was to evaluate the level of awareness among this demographic, which is particularly susceptible to IHD.

### Study population and sampling

Participants were selected using a non-probability convenience sampling method, ensuring a diverse representation from various regions. Inclusion criteria were set to include individuals aged 60 years and older who were residents of Egypt. Exclusion criteria included healthcare professionals, individuals who declined to participate, and those who did not complete the survey.

The minimum required sample size for the study was calculated using the Raosoft sample size calculator with the following parameters: a population proportion of 50%, a margin of error of 5%, and a confidence interval (CI) of 95%. Using the formula: n = (Z 2 * *p* * (1—*p*)) / e 2. Where Z is 1.96 (corresponding to a 95% confidence level), *p* is 0.5 (the assumed population proportion), and e is 0.05 (the margin of error), the minimum required sample size was determined to be 384 participants. To ensure the robustness of the results and account for potential dropouts and non-responses, we targeted a minimum of 500 participants for this study. The survey was completed by 595 participants. This sample size was deemed sufficient to achieve a statistically significant analysis of IHD awareness in the elderly population.

### Data collection instrument

Data for this study were collected using a structured, self-administered questionnaire designed specifically for the elderly population in Egypt. The questionnaire was adapted from a previously validated instrument used in a study conducted in rural Tanzania, which assessed public knowledge of cardiovascular disease (CVD) risk factors and warning signs [9]. To ensure cultural relevance and linguistic accuracy, the questionnaire was translated into Arabic and then back-translated into English to verify consistency. The questionnaire was then piloted with 50 elderly participants to assess its clarity and appropriateness in the Egyptian context. Feedback from the pilot study led to refinements that enhanced the questionnaire's suitability for this population.

The final version of the questionnaire comprised two main sections. The first section gathered sociodemographic data, including age, gender, marital status, educational level, occupation, economic status, and residence (urban or rural). The second section focused on the assessment of knowledge related to ischemic heart disease (IHD). Participants were asked to identify 12 risk factors, and 9 warning signs associated with IHD. Knowledge levels were categorized into four groups: good (scores > 7), moderate (scores 4–7), poor (scores < 4), and no knowledge (score of 0). This structured approach allowed for a comprehensive assessment of IHD awareness among the elderly population in Egypt.

### Pilot study and validation

A pilot study was carried out to rigorously test and refine the questionnaire before its full deployment. This preliminary phase involved administering the survey to a sample of 50 elderly participants representative of the study's target population. The primary objectives of the pilot study were to assess the clarity, comprehensibility, and relevance of the questionnaire items, as well as to identify any potential ambiguities or cultural misalignments. Participants were encouraged to provide detailed feedback on the survey’s content, including the ease of understanding the questions, the appropriateness of the language used, and the overall length of the questionnaire. Based on the feedback received, several modifications were made to enhance the questionnaire's usability and effectiveness. These adjustments included rephrasing certain questions for greater clarity, simplifying complex terminologies, and ensuring that the content was culturally sensitive and relevant to the Egyptian elderly population. Additionally, the layout and format of the questionnaire were optimized to facilitate easier navigation for participants.

The reliability of the knowledge assessment scales within the questionnaire was then evaluated through statistical analysis using Cronbach’s alpha, a measure of internal consistency. The resulting Cronbach’s alpha value of 0.827 indicated a high level of reliability, confirming that the questionnaire was a robust tool for assessing knowledge of ischemic heart disease risk factors and warning signs among the study population. This validation process ensured that the final version of the questionnaire was both scientifically sound and well-suited to the study’s objectives.

### Data collection procedure

Data were collected exclusively through an online survey to ensure broad and efficient reach across Egypt's elderly population. A Google Form link was distributed via various social media platforms, including Facebook, WhatsApp, and local community groups, specifically targeting participants aged 60 years and above. The survey was designed to be easily accessible and user-friendly, allowing participants to complete it at their convenience. The research team monitored the data collection process to maintain data integrity and ensure that responses were submitted correctly.

### Statistical analysis

Data were analyzed using IBM SPSS Statistics for Windows, version 26. Descriptive statistics were employed to summarize the demographic characteristics and knowledge levels of the participants. Continuous variables, which were confirmed to have a non-normal distribution through the Shapiro–Wilk test, were presented as medians and interquartile ranges. Categorical variables were summarized using frequencies and percentages to provide a clear overview of the distribution of responses across different demographic and knowledge categories. For comparative analyses, non-parametric tests were utilized due to the non-normal distribution of the data. The Mann–Whitney U test was used to compare knowledge scores between two groups, allowing for the identification of significant differences in median scores. The Kruskal–Wallis test was applied when comparing knowledge scores across more than two groups, facilitating the analysis of variations across multiple demographic factors. In addition to these statistical analyses, Python was utilized for the creation of figures and visual representations of the data. The use of Python allowed for precise and customizable visualizations, enhancing the clarity and impact of the presented data in the study. A two-sided *P*-value of less than 0.05 was considered statistically significant, indicating a meaningful difference between groups.

**Ethical considerations.**

The study was conducted in accordance with the ethical principles outlined in the Declaration of Helsinki and received approval from the Mansoura Faculty of Medicine Institutional Research Board (IRB) (Approval No. R.23.10.2355). Informed consent was obtained from all participants prior to data collection. For online participants, consent was embedded within the Google Form, and for face-to-face interactions, verbal consent was recorded by the interviewer. Participants were assured of the confidentiality of their responses, and all data were anonymized before analysis.

## Results

### Participant demographics and characteristics

The sample comprised 595 elderly individuals, with a male predominance of 54.3% (n = 323). The residence distribution showed a slight majority from rural areas (53.8%, n = 320). Marital status varied significantly, with most participants being married (76.3%, n = 454), followed by widowed (15.5%, n = 92), divorced (5.7%, n = 34), unmarried (1.5%, n = 9), and separated (1.0%, n = 6). Educational levels among participants indicated that 30.4% (n = 181) had no formal education. The economic status revealed that the majority were at a middle level (51.1%, n = 304). In terms of occupation, housewives comprised the largest group (25.9%, n = 154), followed by retirees (20.5%, n = 122). Smaller occupational groups included farmers (10.8%, n = 64), teachers (6.2%, n = 37), unemployed individuals (3.0%, n = 18), engineers (2.2%, n = 13), and others (2.7%, n = 16). Height and weight distributions indicated that 40.8% (n = 243) of participants were 160–169 cm tall and 35.5% (n = 211) weighed 80–89 kg. Table 1 provides a detailed overview of the demographic and characteristic data of the study participants.

**Table 1:** Demographics and characteristics of the participants

Characteristics	Frequency	%
*Gender*		
Male	323	54.3
Female	272	45.7
*Residence*		
Urban	275	46.2
Rural	320	53.8
*Marital status*		
Unmarried	9	1.5
Married	454	76.3
Divorced	34	5.7
Widower	92	15.5
Separate	6	1.0
*Educational level*		
N/A	181	30.4
Primary	36	6.1
Preparatory	37	6.2
Secondary	99	16.6
University	165	27.7
Postgraduate	77	12.9
*Economic level*		
Bad	65	10.9
Good	175	29.4
Middle	304	51.1
Excellent	51	8.6
*Occupation*		
Housewife	154	25.9
Employee/Worker	103	17.3
Engineer	13	2.2
Business/Commerce	68	11.4
Unemployed	18	3.0
Farmer	64	10.8
Teacher	37	6.2
Retired	122	20.5
Others	16	2.7
*Height (cm)*		
Less than 150	10	1.7
150–159	71	11.9
160–169	243	40.8
170–179	180	30.3
180–189	84	14.1
More than 190	7	1.2
*Weight (kg)*		
Less than 50	2	0.3
50–59	10	1.7
60–69	48	8.1
70–79	133	22.4
80–89	211	35.5
More than 90	191	32.1

### Level of knowledge for risk factors, warning signs, and overall, knowledge

Table 2 summarizes the participants' knowledge levels regarding ischemic heart disease. Regarding knowledge of risk factors, 18.7% (n = 111) exhibited good knowledge, 21% (n = 125) had moderate knowledge, and 47.2% (n = 281) had poor knowledge. Additionally, 13.1% (n = 78) reported no knowledge at all about risk factors. Concerning warning signs, only 7.2% (n = 43) demonstrated good knowledge, while 28.1% (n = 167) had moderate knowledge, and 47.9% (n = 285) had poor knowledge. A notable 16.8% (n = 100) of participants had no knowledge of warning signs. Overall knowledge of ischemic heart disease revealed that 15.1% (n = 90) had good knowledge, 22.7% (n = 135) had moderate knowledge, 51.8% (n = 308) had poor knowledge, and 10.4% (n = 62) had no knowledge at all.

**Table 2:** Level of knowledge for risk factors, warning signs, and overall knowledge

Level of knowledge	Risk factors, n (%)	Warning signs, n (%)	Total knowledge, n (%)
Good knowledge	111 (18.7)	43 (7.2)	90 (15.1)
Moderate knowledge	125 (21)	167 (28.1)	135 (22.7)
Poor knowledge	281 (47.2)	285 (47.9)	308 (51.8)
No knowledge at all	78 (13.1)	100 (16.8)	62 (10.4)

**Fig. 1:**
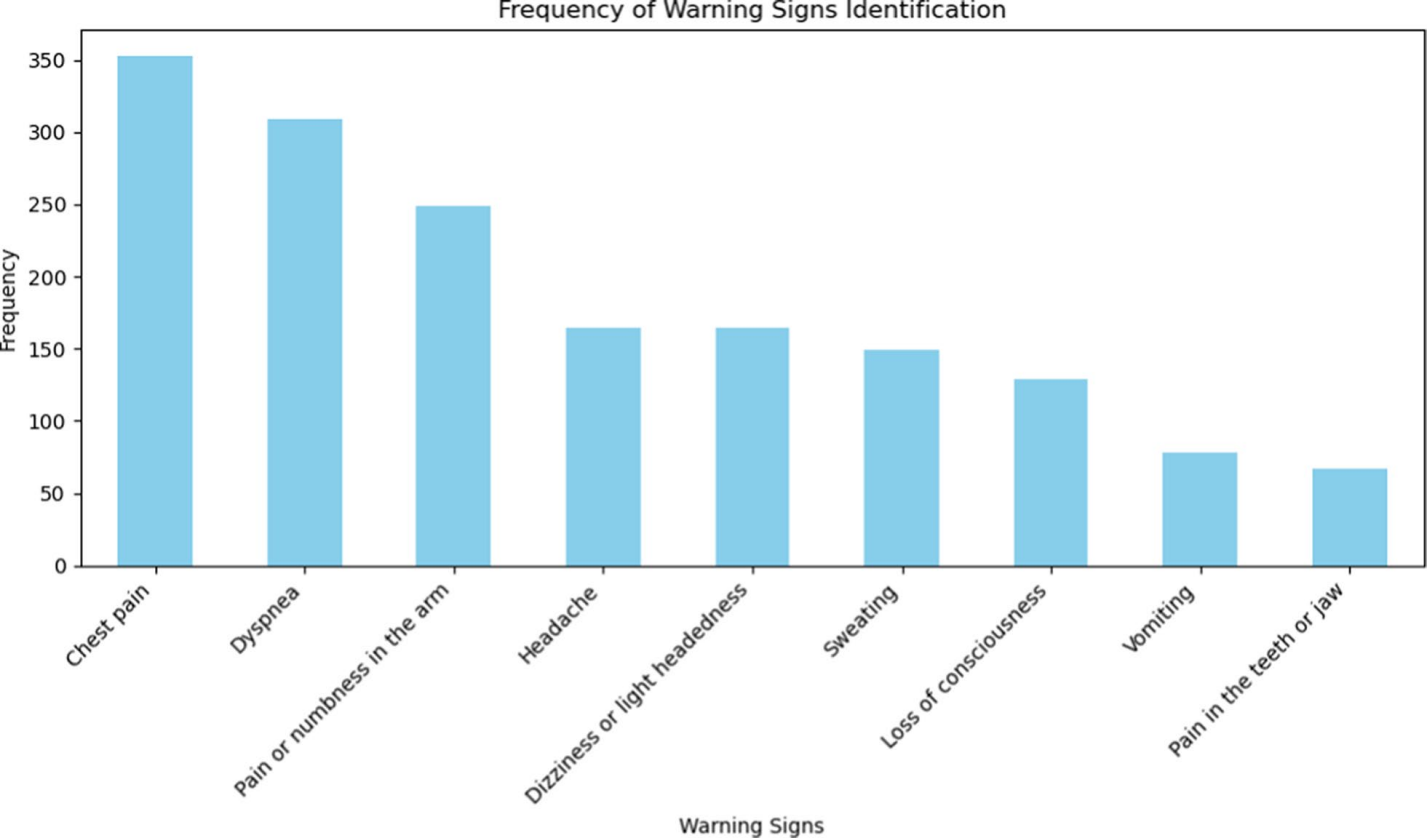
Frequency of warning signs identification

Regarding the identification of warning signs for cardiovascular disease events, the most frequently recognized sign among the participants was chest pain, with approximately 330 individuals identifying it as a key symptom. Following chest pain, dyspnea (shortness of breath) was the second most acknowledged warning sign, noted by around 300 participants. Pain or numbness in the arm ranked third, identified by nearly 250 respondents. Other symptoms, including headaches and dizziness or lightheadedness, were recognized by around 200 participants each. Signs like sweating, loss of consciousness, and vomiting were less frequently mentioned, with fewer than 150 respondents recognizing them. Notably, pain in the teeth or jaw was the least identified warning sign, with only around 60 participants acknowledging it (see Fig. 1).

**Fig. 2:**
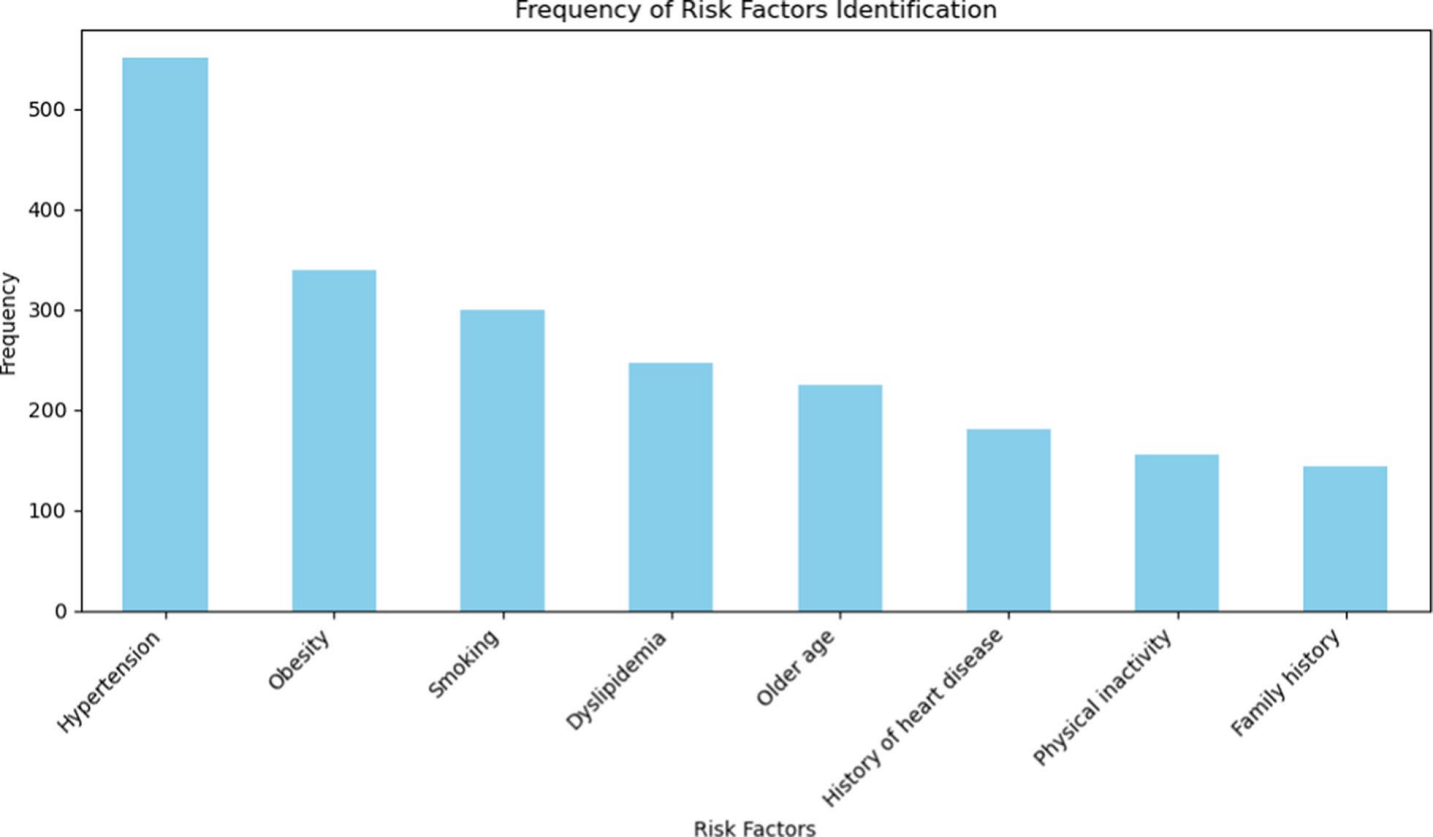
Frequency of Risk Factors identification

In terms of risk factor identification, hypertension was the most recognized risk factor for ischemic heart disease, with over 500 participants acknowledging it. Obesity followed, identified by nearly 400 respondents; while smoking and dyslipidemia were recognized by approximately 350 and 300 participants, respectively. Other risk factors, such as older age, history of heart disease, and physical inactivity, were identified by fewer participants, with less than 200 each. The least recognized risk factor was family history, identified by fewer than 100 participants (see Fig. 2).

### Association between demographic criteria and knowledge

Table 3 explores the association between demographic criteria and knowledge scores related to ischemic heart disease. Gender did not significantly impact knowledge levels, with median risk factors scores being 3.3 for males and 2.5 for females (*p* = 0.8), and warning signs scores of 3.3 for males and 2.2 for females (*p* = 0.07). The total knowledge scores were 2.9 for males and 2.4 for females (*p* = 0.3). Residence significantly influenced knowledge scores, with urban participants scoring higher on risk factors (median: 3.3) compared to rural participants (median: 2.5), with a *p*-value of < 0.05. Urban participants also had higher total knowledge scores (median: 3.8) compared to rural participants (median: 1.9), *p*-value < 0.05. No significant difference was found for warning signs score (*p* = 0.2).

**Table 3:** Association between demographic criteria and knowledge

Demographic Criteria	Risk Factors Score, Median (IQR)	*P*-value	Warning Signs Score, Median (IQR)	*P*-value	Total Score, Median (IQR)	*P*-value
*Gender*		0.8		0.07		0.3
Male	3.3 (10)		3.3 (10)		2.9 (10)	
Female	2.5 (10)		2.2 (10)		2.4 (10)	
*Original Residence*		< 0.05		0.2		< 0.05
Urban	3.3 (10)		3.3 (10)		3.8 (10)	
Rural	2.5 (10)		2.2 (10)		1.9 (10)	
*Educational Level*		< 0.05		< 0.05		< 0.05
N/A	1.7 (10)		1.1 (10)		1.0 (10)	
Primary	0.8 (5.8)		1.1 (7.8)		1.0 (6.7)	
Preparatory	0.8 (10)		1.1 (6.7)		1.0 (8.6)	
Secondary	3.3 (10)		3.3 (10)		2.9 (10)	
University	5.8 (10)		4.4 (10)		5.2 (10)	
Postgraduate	4.2 (10)		3.3 (10)		4.3 (10)	
*Marital Status*		0.1		0.09		0.1
Unmarried	1.7 (5.8)		2.2 (2.2)		1.9 (4.3)	
Married	3.3 (10)		3.3 (10)		2.9 (10)	
Divorced	2.5 (10)		2.8 (10)		2.9 (10)	
Separated	1.7 (4.2)		2.2 (2.2)		1.9 (3.3)	
Widower	1.7 (10)		1.1 (10)		1.4 (10)	
*Economic Level*		< 0.05		< 0.05		< 0.05
Bad	0.8 (10)		1.1 (10)		1.0 (10)	
Middle	2.5 (10)		2.2 (10)		2.4 (10)	
Good	5.8 (10)		4.4 (10)		4.8 (10)	
Excellent	4.2 (10)		4.4 (10)		4.3 (10)	
*Occupation*		< 0.05		< 0.05		< 0.05
Housewife	2.5 (10)		2.2 (10)		2.4 (10)	
Employee/Worker	3.3 (10)		3.3 (10)		3.8 (10)	
Business/Commerce	1.7 (9.2)		2.2 (7.8)		1.9 (8.6)	
Engineer	7.5 (9.2)		4.4 (8.9)		5.7 (9)	
Farmer	0.8 (5.8)		1.1 (5.6)		1.0 (5.2)	
Teacher	5.0 (10)		4.4 (10)		4.8 (10)	
Retired	4.2 (10)		3.3 (10)		4.3 (10)	
Others	5.8 (10)		3.3 (7.8)		5.5 (8.6)	

Educational level had a significant impact on knowledge scores. Postgraduate participants had the highest median scores across all dimensions: risk factors (4.2), warning signs (3.3), and total knowledge (4.3), all with *p*-values < 0.05. Participants with no formal education had the lowest scores: risk factors (1.7), warning signs (1.1), and total knowledge (1.0). Economic status also significantly influenced knowledge levels. Participants in the excellent economic category had higher median scores for risk factors (4.2), warning signs (4.4), and total knowledge (4.3) compared to those in the bad economic category, with median scores of 0.8 for risk factors, 1.1 for warning signs, and 1.0 for total knowledge, all with *p*-values < 0.05. Occupation also had a significant effect on knowledge scores. Engineers had the highest median risk factors score (7.5), warning signs score (4.4), and total knowledge score (5.7), all with *p*-values < 0.05. Housewives had median scores of 2.5 for risk factors, 2.2 for warning signs, and 2.4 for total knowledge, while farmers had the lowest scores. These findings suggest that professional background and occupation influence health knowledge, possibly due to differences in access to health information and educational resources.

### Health concerns among participants

**Fig. 3:**
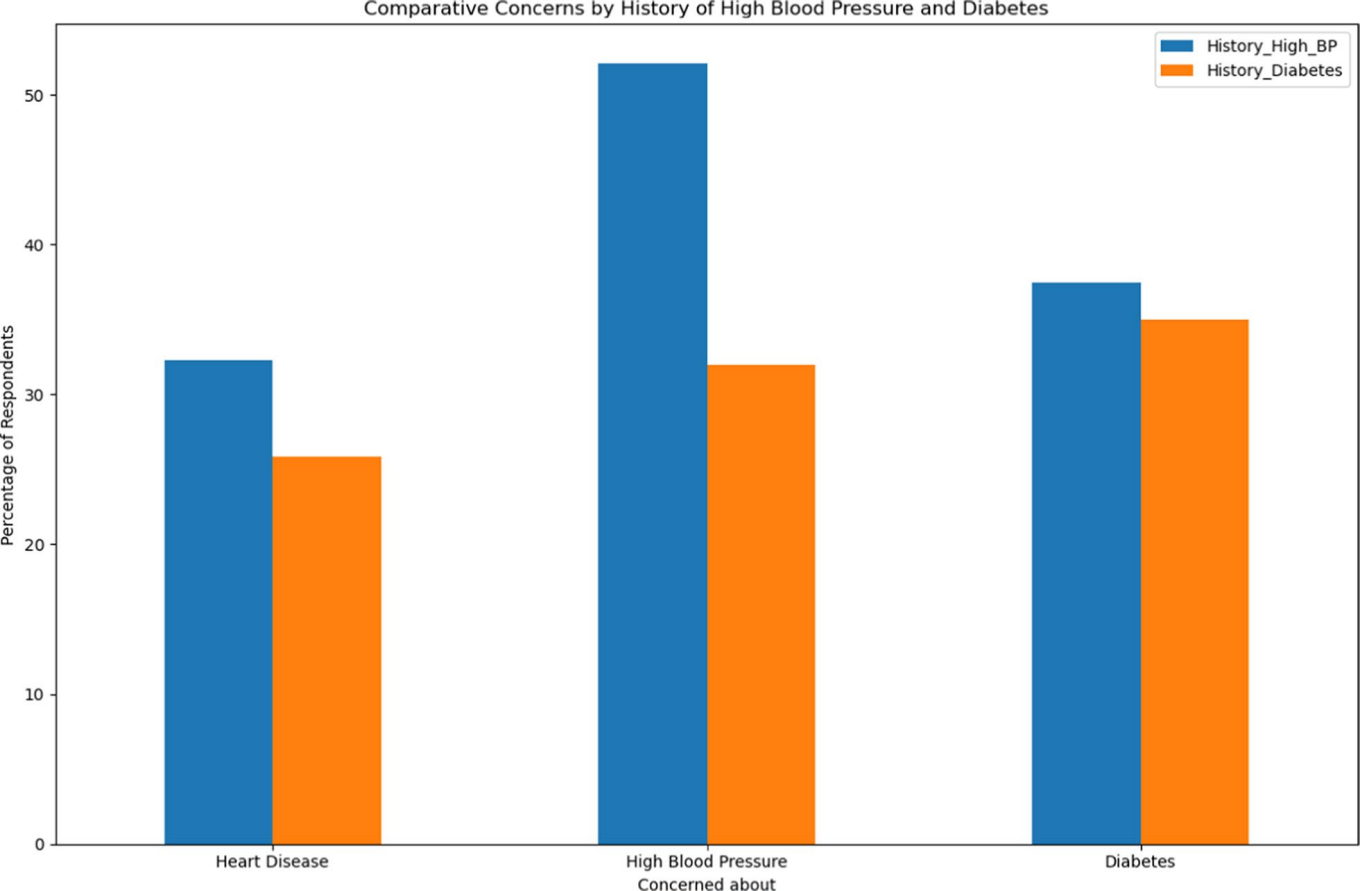
Compare between who have personal history of Diabetes and Hypertension regarding concerns about having Heart diseases, High blood pressure and Diabetes

The survey results highlight the concerns of individuals with a personal history of diabetes and hypertension regarding the development of related conditions. Specifically, 30% of individuals with hypertension and 20% of those with diabetes express concerns about developing heart disease. When it comes to high blood pressure, concerns are higher among those with hypertension, with 50% indicating worry, compared to 35% of participants with diabetes. Interestingly, concerns about diabetes are similar between both groups, with 40% of individuals with diabetes and 35% of those with hypertension expressing worry about developing the condition (Fig. 3).

**Fig. 4:**
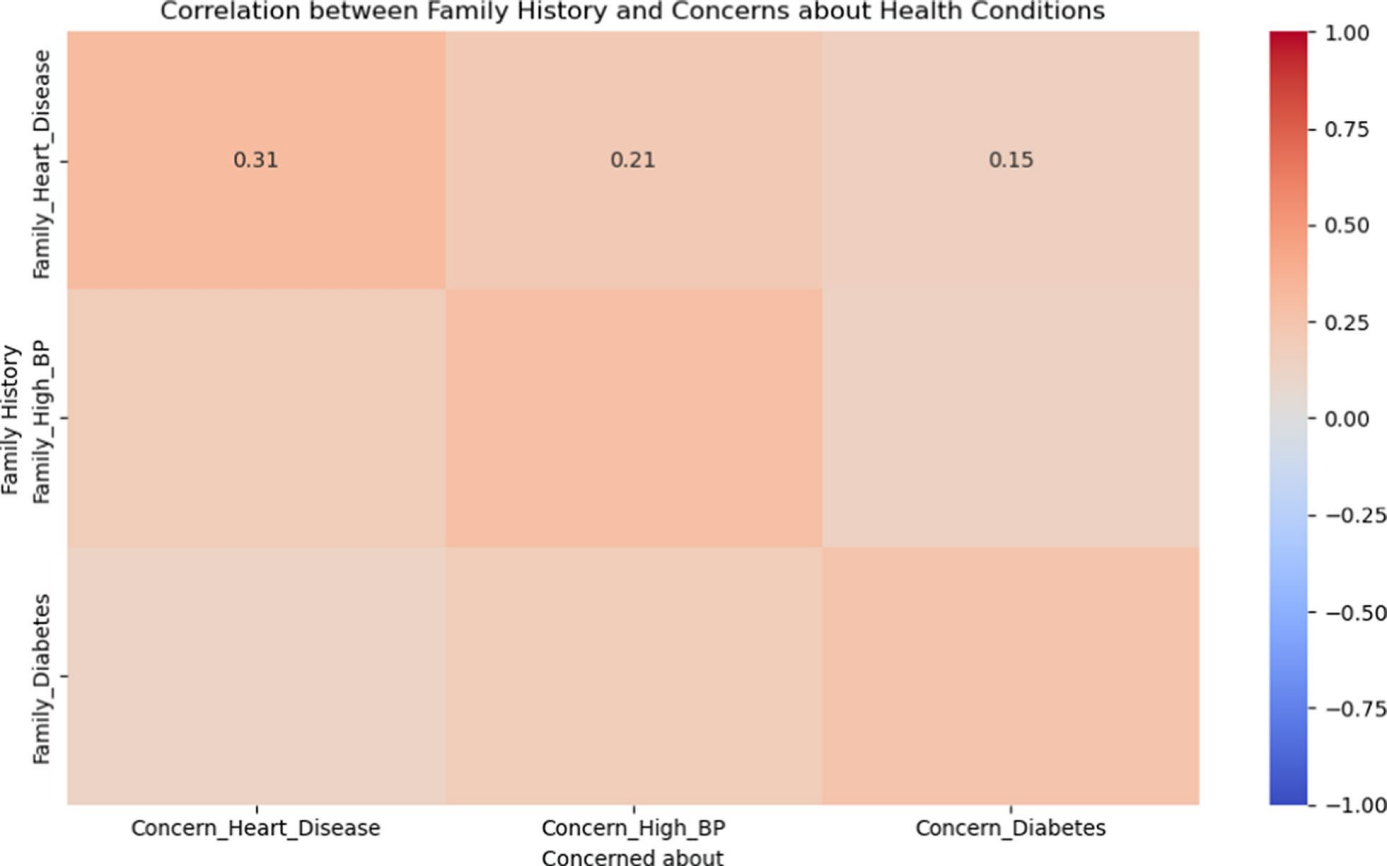
Heatmap showing correlation between Family history and Concerns about health conditions

**Fig. 5:**
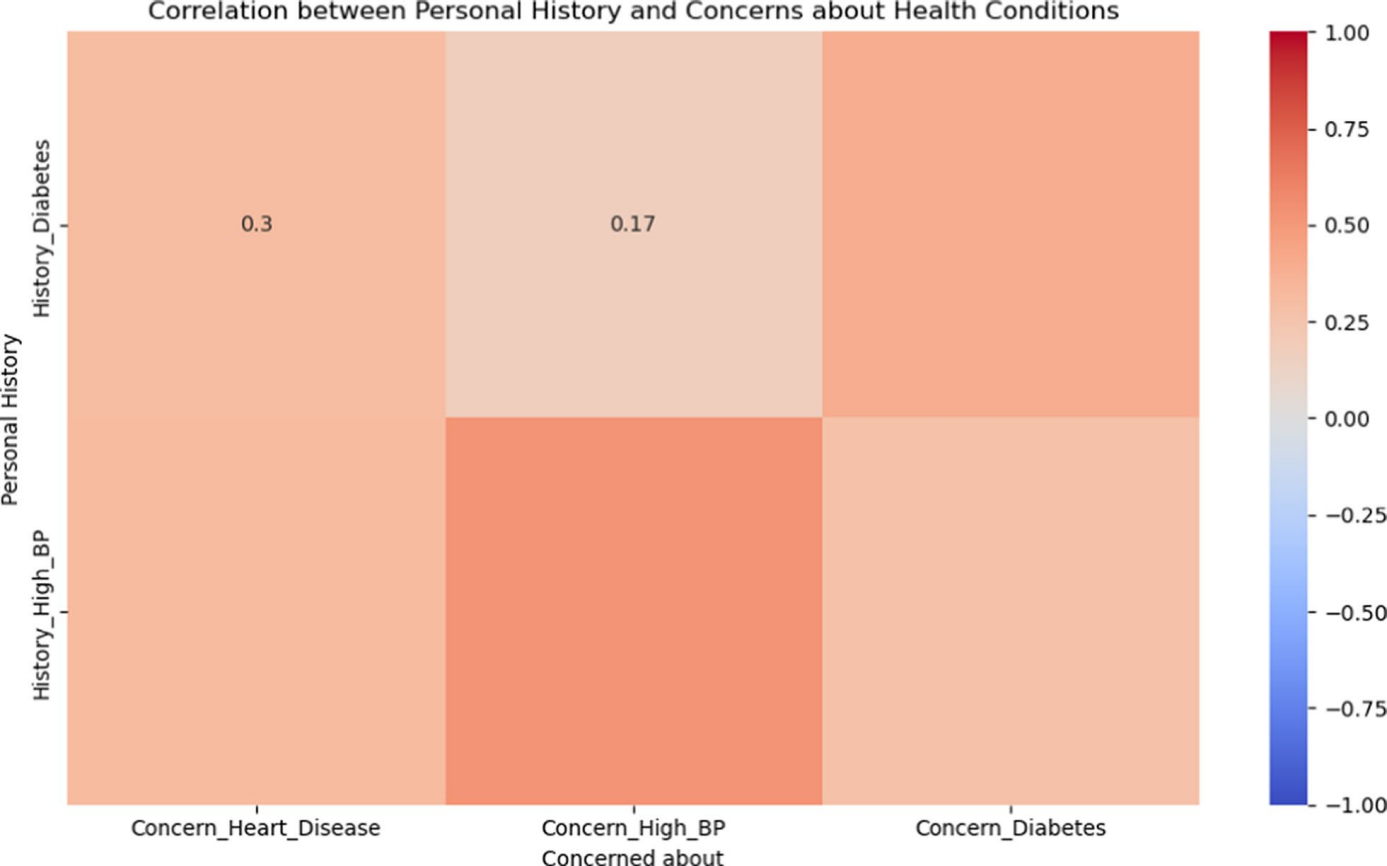
Heatmap showing correlation between Personal history and Concerns about health conditions

A strong correlation is observed between family history and health concerns. For instance, individuals with a family history of heart disease show a strong concern about developing heart diseases themselves (r = 0.75). Similarly, there is a high correlation between family history of diabetes and concern about the condition (r = 0.80). The heatmap also indicates that personal history of diabetes has a high correlation with concerns about diabetes (r = 0.85) and a moderate correlation with concerns about high blood pressure (r = 0.60). Likewise, a personal history of hypertension shows a high correlation with concerns about high blood pressure (r = 0.80) and a moderate correlation with concerns about heart disease (r = 0.65) (Figs. 4 and 5).

## Discussion

### IHD overall knowledge

To the best of our knowledge, this is the first study to comprehensively address the current awareness of risk factors and warning signs of IHD among the elderly in all of Egypt. According to our findings, 62.2% of participants had overall poor to no knowledge of IHD risk factors and warning signs, which reflects the elderly’s low awareness of the causes and symptoms of IHD. Lower levels of awareness were reported in a study conducted in Kuwait, with 66.7% of old-aged participants having overall poor knowledge[10]. Nonetheless, studies from China and Syria revealed higher rates, showing that only 56.9% and 28.3% of old-aged participants had low knowledge of CVD risk factors and warning signs, respectively[7, 9].

According to our findings, hypertension and obesity were the most identified risk factors for IHD. This aligns with a study done in Korea, where hypertension was the most frequently identified risk factor[6]. Obesity and physical inactivity were recognized by around two-thirds of the participants in our study, which matches the results of the Korean study; nevertheless, they were not sufficiently identified among our participants. This could be explained by the elderly's lack of awareness of the importance of leading a healthy, active lifestyle. A 2019 study conducted among the elderly in Egypt found that 71.2% of participants had low physical activity levels, especially those with urban residency[11]. Another recent study found a large decline in outdoor time, a rise in housework, and a drop in physical exercise among the elderly in Egypt after the COVID-19 crisis[12].

Our study found that the most recognized warning signs were chest pain and shortness of breath. These results are inconsistent with a previous Chinese study[7]. The least identified warning sign was pain in the teeth or jaw. Based on our results, IHD risk factors were more widely known than its warning signs. This finding is consistent with studies conducted on the general population in Tanzania, Riyadh, and Kenya [13–15].

### Factors associated with the knowledge of IHD

As predicted, we discovered that higher overall knowledge levels were associated with higher education levels and economic status. These findings match with previous research conducted among vulnerable communities in Belgium and England as well as the general population in rural Tanzania and Ethiopia [13, 16, 17]. Regarding the participant’s occupations, farmers had the lowest knowledge, while engineers had the highest, which may be due to their education level.

Future research should adopt a longitudinal approach to capture changes in ischemic heart disease (IHD) awareness over time, helping clarify the long-term impacts of socioeconomic factors and public health interventions. Expanding sampling to include underserved rural and economically disadvantaged populations is critical for accurate representation, potentially through face-to-face interviews to overcome digital access limitations. Solutions include targeted public health campaigns in rural areas, involving community health workers and trusted local figures to enhance engagement. Multi-channel strategies—social media, radio, and family-involved initiatives—can further improve reach, emphasizing IHD warning signs and proactive health behaviors. Culturally tailored materials, such as visual guides, could simplify complex concepts for elderly audiences. Integrating IHD awareness into routine primary healthcare for the elderly and using mobile health units in rural regions could sustain knowledge and early intervention. These combined efforts are essential for reducing IHD burden among Egypt’s aging population.

### Limitations

This study has several limitations that should be acknowledged. First, the cross-sectional design provides a snapshot of ischemic heart disease (IHD) awareness among Egypt’s elderly population but does not establish causality. This limits our ability to draw conclusions about the directionality or long-term impacts of factors influencing awareness, such as education and economic status. A longitudinal approach might yield more insights into the evolving awareness and knowledge over time. Second, the use of convenience sampling and an online survey format may have introduced selection bias, potentially favoring individuals with better digital literacy or access to technology, which might skew representation. Older adults with limited internet access, particularly those in lower economic strata or remote rural areas, may have been underrepresented, potentially leading to an overestimation of the general awareness level. Additionally, self-reported data, as collected through online surveys, are inherently susceptible to response and recall bias, possibly impacting the accuracy of participants’ reported knowledge levels and health concerns. The questionnaire itself, though piloted and validated, was adapted from an instrument used in a different cultural context (rural Tanzania). While efforts were made to ensure its relevance to the Egyptian elderly population, subtle cultural and contextual differences might still have influenced participants' interpretations and responses, affecting the reliability of the findings. Lastly, while non-parametric tests were used to account for the non-normal distribution of data, the demographic diversity within certain subgroups, such as specific occupations or economic categories, was limited. This restricts the generalizability of the findings, particularly concerning the associations between occupation and IHD knowledge levels.

## Conclusion

In conclusion, this study reveals a substantial lack of awareness about ischemic heart disease (IHD) risk factors and warning signs among the elderly in Egypt, with 62.2% showing poor or no knowledge. Higher education and economic status were associated with better awareness, highlighting the need for targeted interventions. Large-scale awareness campaigns focusing on vulnerable populations are necessary to improve knowledge and promote healthier lifestyles. Further research is needed to assess the impact of these efforts on IHD outcomes.

## Supplementary Information

Additional file1

## Data Availability

The datasets used during the current study are available from the corresponding author upon reasonable request.

## Abbreviations

**IHD**: Ischemic Heart Disease
**CVD**: Cardiovascular Disease
**EMR**: Eastern Mediterranean Region
**CI**: Confidence Interval
**IRB**: Institutional Research Board
**SPSS**: Statistical Package for the Social Sciences
