# Supplementary material for: Ischemic heart disease awareness in Egypt's aging population: findings from a national cross-sectional study
Source: Egypt Heart J. 2024 Nov 22;76:152. doi: 10.1186/s43044-024-00584-1 (PMC11584834; doi:10.1186/s43044-024-00584-1)
Supplement: Supplementary file 1 — Additional file1 [file 43044_2024_584_MOESM1_ESM.doc]

STROBE Statement—Checklist of items that should be included in reports of ***cross-sectional studies***

|  | Item No | Recommendation | Reported on Page/Section |
| --- | --- | --- | --- |
| **Title and abstract** | 1 | (*a*) Indicate the study’s design with a commonly used term in the title or the abstract | Title Page, Abstract |
|  |  | (*b*) Provide in the abstract an informative and balanced summary of what was done and what was found | Abstract, Page 1 |
| Introduction | | |  |
| Background/rationale | 2 | Explain the scientific background and rationale for the investigation being reported | Introduction, Background, Page 2, Paragraph 1-3 |
| Objectives | 3 | State specific objectives, including any prespecified hypotheses | Introduction, Objectives, Page 2, Paragraph 4 |
| Methods | | |  |
| Study design | 4 | Present key elements of study design early in the paper | Methods, Study Design, Page 3, Paragraph 1 |
| Setting | 5 | Describe the setting, locations, and relevant dates, including periods of recruitment, exposure, follow-up, and data collection | Methods, Setting, Page 3, Paragraph 2 |
| Participants | 6 | (*a*) Give the eligibility criteria, and the sources and methods of selection of participants | | Methods, Participants, Page 3, Paragraph 3 | | --- |  |  | | --- | |
| Variables | 7 | Clearly define all outcomes, exposures, predictors, potential confounders, and effect modifiers. Give diagnostic criteria, if applicable | | Methods, Variables, Page 4, Paragraph 1 | | --- |  |  | | --- | |
| Data sources/ measurement | 8* | For each variable of interest, give sources of data and details of methods of assessment (measurement). Describe comparability of assessment methods if there is more than one group | | *Methods, Data Collection Instrument, Page 4, Paragraph 2* | | --- |  |  | | --- | |
| Bias | 9 | Describe any efforts to address potential sources of bias | | Methods, Bias, Page 4, Paragraph 3 | | --- |  |  | | --- | |
| Study size | 10 | Explain how the study size was arrived at | | Methods, Study Size, Page 4, Paragraph 4 | | --- |  |  | | --- | |
| Quantitative variables | 11 | Explain how quantitative variables were handled in the analyses. If applicable, describe which groupings were chosen and why | | Methods, Quantitative Variables, Page 5, Paragraph 1 | | --- |  |  | | --- | |
| Statistical methods | 12 | (*a*) Describe all statistical methods, including those used to control for confounding | Methods, Statistical Analysis, Page 5, Paragraph 2 |
|  |  | (*b*) Describe any methods used to examine subgroups and interactions | Methods, Statistical Methods, Page 5, Paragraph 2 |
|  |  | (*c*) Explain how missing data were addressed | Methods, Statistical Methods, Page 5, Paragraph 2 |
|  |  | (*d*) If applicable, describe analytical methods taking account of sampling strategy |  |
|  |  | (*e*) Describe any sensitivity analyses | \| Methods, Statistical Methods, Page 5, Paragraph 4 \| \| --- \|  \|  \| \| --- \| |
| Results | | |  |
| Participants | 13* | (a) Report numbers of individuals at each stage of study—eg numbers potentially eligible, examined for eligibility, confirmed eligible, included in the study, completing follow-up, and analysed | | Methods, Statistical Methods, Page 5, Paragraph 5 | | --- |  |  | | --- | |
|  |  | (b) Give reasons for non-participation at each stage | \| Results, Participant Flow, Page 6, Paragraph 1 \| \| --- \|  \|  \| \| --- \| |
|  |  | (c) Consider use of a flow diagram | \| \| N/A \| \| --- \|  \|  \| \| --- \| \| \| --- \| --- \| --- \|  \|  \| \| --- \| |
| Descriptive data | 14* | (a) Give characteristics of study participants (eg demographic, clinical, social) and information on exposures and potential confounders | | Results, Descriptive Data, Page 6, Paragraph 3 | | --- |  |  | | --- | |
|  |  | (b) Indicate number of participants with missing data for each variable of interest | \| Results, Descriptive Data, Page 6, Paragraph 4 \| \| --- \|  \|  \| \| --- \| |
| Outcome data | 15* | Report numbers of outcome events or summary measures | Results, Outcome Data, Page 7, Paragraph 1 |
| Main results | 16 | (*a*) Give unadjusted estimates and, if applicable, confounder-adjusted estimates and their precision (eg, 95% confidence interval). Make clear which confounders were adjusted for and why they were included | | Results, Main Results, Page 7, Paragraph 2 | | --- |  |  | | --- | |
|  |  | (*b*) Report category boundaries when continuous variables were categorized | \| Results, Main Results, Page 7, Paragraph 3 \| \| --- \|  \|  \| \| --- \| |
|  |  | (*c*) If relevant, consider translating estimates of relative risk into absolute risk for a meaningful time period | \| N/A \| \| --- \|  \|  \| \| --- \| |
| Other analyses | 17 | Report other analyses done—eg analyses of subgroups and interactions, and sensitivity analyses | Results, Other Analyses, Page 7, Paragraph 4 |
| Discussion | | |  |
| Key results | 18 | Summarise key results with reference to study objectives | Discussion, Key Results, Page 8, Paragraph 1 |
| Limitations | 19 | Discuss limitations of the study, taking into account sources of potential bias or imprecision. Discuss both direction and magnitude of any potential bias | Discussion, Limitations, Page 8, Paragraph 2 |
| Interpretation | 20 | Give a cautious overall interpretation of results considering objectives, limitations, multiplicity of analyses, results from similar studies, and other relevant evidence | Discussion, Interpretation, Page 8, Paragraph 3 |
| Generalisability | 21 | Discuss the generalisability (external validity) of the study results | Discussion, Generalisability, Page 8, Paragraph 4 |
| Other information | | |  |
| Funding | 22 | Give the source of funding and the role of the funders for the present study and, if applicable, for the original study on which the present article is based | Other Information, Funding, Page 9, Paragraph 1 |

*Give information separately for exposed and unexposed groups.

**Note:** An Explanation and Elaboration article discusses each checklist item and gives methodological background and published examples of transparent reporting. The STROBE checklist is best used in conjunction with this article (freely available on the Web sites of PLoS Medicine at http://www.plosmedicine.org/, Annals of Internal Medicine at http://www.annals.org/, and Epidemiology at http://www.epidem.com/). Information on the STROBE Initiative is available at www.strobe-statement.org.
